# Extracting activity of individual cell populations from multielectrode recordings

**DOI:** 10.1186/1471-2202-12-S1-P374

**Published:** 2011-07-18

**Authors:** Jan Potworowski, Helena Głąbska, Szymon Łęski, Daniel K Wójcik

**Affiliations:** 1 Laboratory of Neuroinformatics, Dept. of Neurophysiology, Nencki Institute, 02-093, Poland

## 

The low-frequency part of the extracellular electric signals, the local field potentials (LFP), carries information about dendritic processing in neuronal populations. However, the long-range nature of electric field makes the analysis of LFP difficult, as typically an electrode records activity of many sources. Modern multielectrodes allow for increased spatial resolution, hence also the need for effective data analysis methods which would allow to get more insight. These methods include Current Source Density (CSD) analysis and source separation methods.

**Figure 1:**
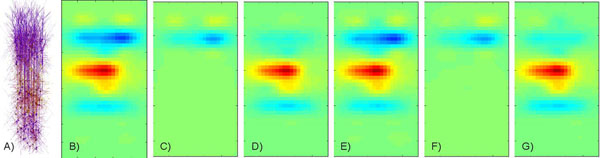
A) Model setup. B) Reconstructed CSD generated in a thalamo-cortical column 20 ms from the onset of stimulation. C) Activity of superior pyramidal cells (rhythmic spiking) in layer 2/3. D) Activity of tufted pyramids (intrinsically bursting) from layer 5. E) sum of two most prominent ICA components shown in F) and G) separately.

In [1] we have combined inverse CSD method [2] with Independent Component Analysis (ICA) [3] to decompose activity recorded in the rat forebrain on a grid of 140 positions obtaining physiologically plausible components across a group of seven animals. The question remains how the obtained components are connected to the activity of neuronal populations. To study this problem we enriched the thalamocortical model [4,5] by adding the spatial information (Figure 1A) and used it to simulate the LFP generated by a single cortical column. We used the kernel CSD method and spatio-temporal ICA to decompose the LFP measured on a regular grid. We compared the resulting components to the activity of the twelve cortical populations included in the model. We found that the recorded evoked activity was dominated by two populations of pyramidal neurons, which were well separated by ICA (Figure 1B-1G). The activity from other populations was hardly visible on top of the main two dipoles and we were also not able to obtain them through ICA.
